# 2D Metalorganic Ferromagnets

**DOI:** 10.1002/advs.202415266

**Published:** 2025-03-05

**Authors:** Egzona Isufi Neziri, Céline Hensky, Hien Quy Le, Diego Radillo Ochoa, Aleksandra Cebrat, Manfred Parschau, Karl‐Heinz Ernst, Christian Wäckerlin

**Affiliations:** ^1^ Molecular Surface Science Group Empa, Swiss Federal Laboratories for Materials Science and Technology Dübendorf CH‐8600 Switzerland; ^2^ Institute of Physics Swiss Federal Institute of Technology Lausanne (EPFL) Station 3 Lausanne CH‐1015 Switzerland; ^3^ Laboratory for X‐ray Nanoscience and Technologies Paul‐Scherrer‐Institut (PSI) Villigen CH‐5232 Switzerland; ^4^ Department of Chemistry University of Zurich Zürich CH‐8057 Switzerland; ^5^ Nanosurf Laboratory Institute of Physics The Czech Academy of Sciences Prague 16200 Czech Republic

**Keywords:** ferromagnetism in low dimensions, scanning probe microscopy, single‐layer low‐dimensional metalorganics

## Abstract

Low‐dimensional materials exhibiting stable magnetic ordering are interesting from a fundamental point of view as well as for future application in information technologies. Metalorganic magnets, created by linking metal atoms with specific organic molecules, allow adjustments to their properties by synthetically modifying the structure of these molecules or the way they connect to the metal atoms. Here, the study details the formation, structure, and magnetic behavior of a single‐layer 2D metalorganic coordination network made of Ni atoms and tetracyanoethylene (TCNE) molecules (2D Ni‐TCNE). Single‐layer crystal domains of this 2D material are achieved by codeposition Ni atoms and TCNE on a Au(111) surface kept in vacuum. Non‐contact atomic force microscopy visualizes the structure with atomic resolution. X‐ray magnetic circular dichroism establishes the 2D NiTCNE as a ferromagnet, with high magnetic remanence and a coercive field of ≈1 tesla at 3 kelvin. The Curie temperature is between 10 and 20 kelvin. Metalorganic chemistry opens a large variety of routes of synthesis and it is anticipated that this materials research paves the way to new magnetic nanomaterials for spintronic applications.

## Graphical abstract

The single‐layered 2D metalorganic coordination network Ni‐TCNE, made from Ni atoms and tetracyanoethylene molecules on a gold surface, is established as a ferromagnet with a high magnetic remanence and coercive field. Its Curie temperature is between 10 and 20 kelvin.

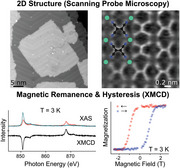

## 1 Introduction

Single‐layer metalorganic magnets^[^ ^1^, ^2^ ^]^ offer promising applications as magnetic semiconductors^[^ ^3^ ^]^ and as model‐systems to rationalize and develop future quantum materials. Since single layers require only minimal amounts of material, they make these substances highly resource‐efficient. Reports on ferromagnetism in low‐dimensional materials, such as organometallic lanthanide compounds,^[^ ^4^ ^]^ van‐der‐Waals materials such as CrI_3_^[^ ^5^ ^]^ as well as stacked and single‐layer metalorganics,^[^ ^6^, ^7^, ^8^ ^]^ and organometallics demonstrate the possibility to establish long‐range magnetic ordering in low‐dimensional hybrid materials. Recently, low‐dimensional metalorganic magnets have been proposed as materials platform that could form the basis for new quantum technology^[^ ^9^, ^10^, ^11^ ^]^ and they have raised significant interest exhibiting exotic magnetic quantum phenomena such as exchange bias^[^ ^12^ ^]^ spin‐liquids^[^ ^13^ ^]^ and potentially Haldane phases.^[^ ^9^ ^]^

**Figure 1:**
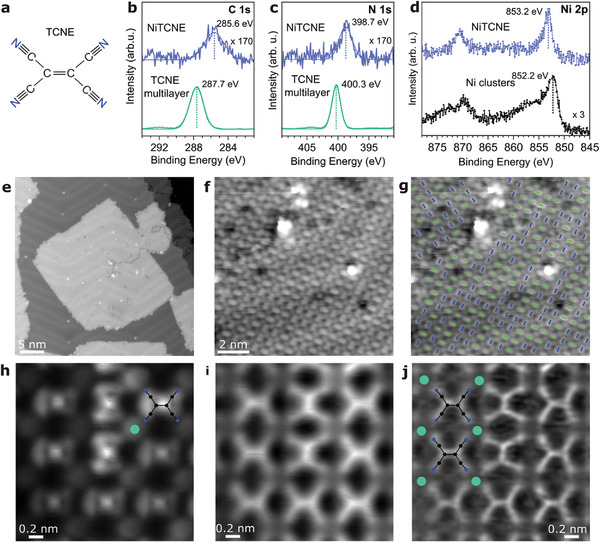
XPS and SPM data of the 2D Ni‐TCNE on Au(111). a) Structure of TCNE. b–d) C 1s, N 1s, and Ni 2p XPS of single‐layer Ni‐TCNE compared with b,c) a multilayer of TCNE molecules and d) metallic Ni impurities on Au(111). The C 1s and N 1s binding energies of Ni‐TCNE are lowered compared to the one of the TCNE reference while the Ni 2p_3/2_ binding energy is increased with respect to metallic Ni. These chemical shifts univocally demonstrate the charge transfer from Ni to TCNE. e–j) Overview STM images (e–g) and constant height STM (h)/AFM (i) scans recorded with a carbon monoxide functionalized probe. Laplace filtered version of the j) AFM image to aid with the identification of the molecular structure. Imaging parameters reported in Table S1 (Supporting Information). Within the essentially square lattice defined by the Ni atoms (d_Ni−Ni_ = 0.67 nm), TCNE adopts two orientations (annotated in (g) with blue and green lines). The Ni atoms are best seen in the STM contrast (h) as round protrusions (annotated as green disks). i,j) Four TCNE molecules coordinate one Ni atom with their cyano‐groups, best seen in the AFM data.

In bulk form, the complexes of the strong electron acceptors tetracyanoquinodimethane (TCNQ) and the structurally similar but smaller TCNE (**Figure** **1**a) were the foundation for collective magnetism in organic and metalorganic materials.^[^ ^14^, ^15^ ^]^ The ferromagnetic cyanocarbon V(TCNE)_2_, discovered more than 30 years ago, exhibits the highest estimated characteristic temperature recorded for any coordination compound in the solid state to date.^[^ ^16^ ^]^

At interfaces, 2D metalorganic layers based on TCNQ and 1,2,4,5‐tetracyanobenzene (TCNB) have been explored with a range of transition‐metals for their coordination chemistry^[^ ^17^, ^18^ ^]^ and collective magnetism.^[^ ^19^, ^20^, ^21^, ^22^ ^]^ Interestingly, 2D Ni‐TCNQ,^[^ ^19^, ^20^, ^21^ ^]^ and 2D Mn‐TCNB^[^ ^22^ ^]^ are superparamagnetic, i.e., they exhibit a high magnetic susceptibility indicating the presence of ferromagnetic coupling, but without stable magnetic ordering. The present report establishes single‐layer 2D Ni‐TCNE as a true ferromagnet in the ordered state, characterized by magnetic remanence and an open magnetic hysteresis loop. Its atomic‐level structure is resolved with scanning probe microscopy (SPM) and the chemical states of Ni‐TCNE are characterized by in situ X‐ray photoelectron spectroscopy (XPS). The magnetic properties are determined by X‐ray absorption spectroscopy (XAS) and X‐ray magnetic circular dichroism (XMCD) measurements.

## 2 Results and Discussion

Single‐layer 2D Ni‐TCNE is fabricated by on‐surface synthesis under ultrahigh vacuum (UHV) on an inert Au(111) substrate. Specifically, well‐ordered 2D crystals of Ni‐TCNE are produced by codeposition of Ni atoms and TCNE molecules on the substrate kept at slightly elevated temperatures (see experimental section/methods for details). The used preparation protocol, where the substrate temperature during nickel and TCNE codeposition is above the desorption temperature of TCNE allows to use the ligand in excess, thereby suppressing the presence of uncoordinated, metallic nickel and at the same time avoiding having leftover unreacted TCNE molecules. Possibly due to this preparation, non‐unity metal/molecules stoichiometries,^[^ ^18^ ^]^ were not observed.

### 2.1 Chemical Characterization by X‐Ray Photoelectron Spectroscopy

2D Ni‐TCNE has C 1s and N 1s signals with binding energies of 285.6 and 398.7 eV, respectively, and has its Ni 2p_3/2_ signal at ∼853.2 eV (Figure 1b–d). Compared to pure TCNE, the N 1s and C 1s peaks are shifted toward lower binding energies. On the other hand, the Ni 2p_3/2_ signal is shifted to higher binding energies compared to metallic Ni. The shift to higher binding energy for nickel and to lower binding energies for carbon and nitrogen implies charge transfer from the metal atoms to TCNE, consistent with data obtained for its bulk analog.^[^ ^23^, ^24^ ^]^

### 2.2 Molecular Structure Revealed by Scanning Probe Microscopy

In overview STM scans (Figure 1e–g), well‐ordered domains exhibiting a square lattice extending tens of nanometers are observed. The good 2D crystalline order is confirmed by sharp peaks in the Fourier‐transform of the STM data (Figure S1, Supporting Information). In constant height STM mode with a carbon monoxide modified tip, the TCNE molecules are imaged as dumbbell‐shaped entities (Figure 1h). The Ni atoms are imaged as round protrusions (Figure 1h). The concurrently performed atomic force microscopy (AFM) is highly sensitive to Pauli repulsion.^[^ ^25^, ^26^ ^]^ Thus, the frequency shift image (Figure 1i) reveals well the molecular structure of TCNE. The visibility of the molecular backbone is further enhanced in the Laplace filtered version of the AFM data (Figure 1j). The nickel atoms are not observed in the AFM contrast (Figure 1i,j) as bright protrusions because they are located a fraction of an angstrom below the plane defined by the TCNE molecules or because they are slightly positively charged, thereby interacting less repulsive with the CO probe particle.

The analysis of the SPM data reveals that the nickel atoms are arranged in an essentially square lattice (Ni − Ni distance: 0.67 nm) by coordination with four cyano‐groups from four TCNE molecules each. However, although TCNE has a twofold C_2_ axis, it adopts two 90°‐rotated orientations at seemingly random positions without apparent distortion of the nickel lattice (Figure 1g).

### 2.3 Magnetic Properties Determined by X‐Ray Magnetic Circular Dichroism

**Figure 2:**
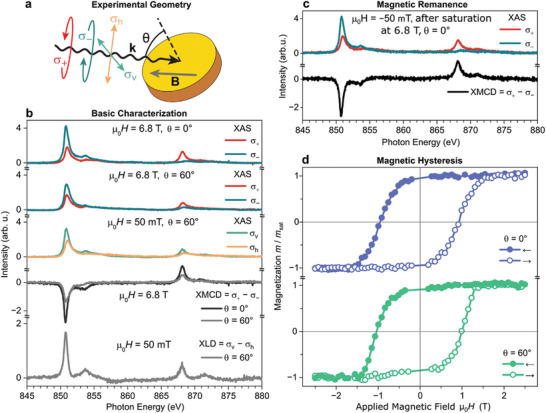
XAS/XMCD/XLD characterization of 2D Ni‐TCNE on Au(111), magnetic remanence, and open magnetic hysteresis. Data recorded at a sample temperature of 3 K on the Ni L_3,2_ edges. a) Sketch of the experimental geometry and definition of the X‐ray polarization: σ_+_ and σ_–_ denote spectra recorded with right and left circularly polarized light; σ_v_ and σ_h_ correspond to linearly polarized light (vertical and horizontal). θ denotes the x‐ray incidence angle with respect to the surface normal. The applied magnetic field is co‐linear with the direction of X‐ray incidence. b) XAS/XMCD at high magnetic field (magnetic saturation) and XAS/XLD that identifies structural ordering. c) XMCD recorded at –50 mT following magnetic saturation at 6.8 T demonstrates remanent magnetization with unchanged sign. d) XMCD magnetization curves (sweep rate: 0.75 T min^−1^; “←”: sweep from 2.5 to –2.5 T; “→”: sweep from –2.5 to 2.5 T) revealing magnetic hysteresis.

X‐ray magnetic circular dichroism (XMCD), which is the difference in X‐ray absorption spectroscopy (XAS) for right and left circularly polarized light (σ_+_ and σ_−_), is a direct measure of the sample magnetization projected on the beam direction. Given the high surface sensitivity of XAS/XMCD in this set‐up, it is ideally suited to reveal the magnetic properties of our single‐layer 2D Ni‐TCNE system. **Figure** **2** presents XAS/XMCD data of 0.74 monolayer (ML) of 2D Ni‐TCNE on Au(111), obtained at the Ni L_3,2_ edges at 3 K. The experimental geometry and X‐ray polarization modes are sketched in Figure 2a. In these experiments, the external magnetic field is co‐linear with the direction of X‐ray incidence. Hence, the XMCD data recorded in normal (θ = 0°, out‐of‐plane) and grazing (θ = 60°, approximately in‐plane) incidence allows determination of the magnetization of the single‐layer in these two directions.

XAS/XMCD data recorded in normal and grazing incidence at high magnetic field (6.8 T) establishes the saturation magnetization (Figure 2b). The XAS/X‐ray linear dichroism (XLD) data shown in Figure 2b serve as a fingerprint of the charge distribution surrounding the Ni atom. The narrow L_3_ line shape and the very strong dependance of XAS on the X‐ray incidence angle are characteristic for a 3d^9^ Ni(I) configuration like in Ni‐TCNQ/Ag(100).^[^ ^19^ ^]^ Furthermore, XA spectra calculated by the multiplet code multiX^[^ ^27^ ^]^ (Figure S3, Supporting Information) fit the XAS/XLD/XMCD spectra well for an assumed 3d^9^ electronic configuration of nickel, i.e., Ni(I). Multiplet calculations using a hypothetical 3d^8^ configuration yield spectra that are incompatible with the experimental data (Figure S3, Supporting Information), hence the more common Ni(II) oxidation state can be firmly excluded. As described in the experimental section, the multiplet calculations also permit to determine the magnetic spin moment <S_z_> from the experimentally measurable value <S_z,eff_> that is obtained by the sum‐rule analysis. The total magnetic moments m_tot_ of 2D Ni‐TCNE at saturation (6.8 T and 3 K) in normal and grazing incidence are identical within the limits of uncertainty (**Table** **1**). This confirms that 2D Ni‐TCNE is magnetically fully saturated in both the out‐of‐plane and the approximately in‐plane direction. In other words, the magnetization has achieved full alignment in both directions. Hence, the magneto‐crystalline anisotropy is small compared to the magnetostatic energy (∼ 2 × m_tot_ × 6.8 T ≈ 1.2 meV).

**Table 1:** Spin (<S_z_>), orbital <L_z_> moments, and total magnetic moments (m_tot_ = (2<S_z_> + <L_z,eff_>)) at saturation (6.8 T, 3 K) as well as coercive fields (H_c_) of NiTCNE. <S_z,eff_> is the effective, uncorrected spin moment obtained by the sum rule analysis.

μ_0_H | T | θ	2<S_z,eff_> [μ_B_]	2<S_z_> [μ_B_]	<L_z,eff_> [μ_B_]	m_tot_ [μ_B_]	μ_0_ H_c_ [T]
**6.8 T | 3 K | 0°**	1.19 ± 0.05	1.28 ± 0.06	0.14 ± 0.03	1.42 ± 0.07	0.95 ± 0.02
**6.8 T | 3 K | 60°**	0.80 ± 0.08	1.46 ± 0.15	0.09 ± 0.05	1.60 ± 0.2	1.01 ± 0.02

### 2.4 Magnetic Remanence

Following the above‐described basic characterization of the magnetic properties of 2D Ni‐TCNE at saturation, the magnetization out of equilibrium is studied. The foremost property of a magnet is its magnetic remanence, i.e., the remaining magnetization in absence of an applied field. Figure 2c shows XAS/XMCD spectra recorded in normal incidence at –50 mT after saturating the magnetization at + 6.8 T. A strong XMCD signal with an unchanged sign compared to saturation is observed. Comparison of the XMCD intensities shows that the remanent magnetization is 93% of the saturation magnetization. The slightly negative applied field excludes any possibility of a spurious remanence due to small residual positive field in the instrument that cannot completely ruled out by ramping the field back to 0 T.

### 2.5 Magnetic Hysteresis

The second important feature of a magnetic material is the presence of a hysteresis in the field dependent magnetization. Such magnetization curves are obtained by recording the field dependent XMCD signal in normal and grazing X‐ray incidence (Figure 2d). Clearly, 2D Ni‐TCNE presents open hysteresis in both measurement geometries. The coercive fields, i.e., external magnetic field that finally coerces the magnetization to change sign, are H_c_ (0°) = 0.95 ± 0.02 T and H_c_ (60°) = 1.01 ± 0.02 T for normal and grazing incidence, respectively (Table 1). The fact that H_c_ (60°) is only slightly larger than H_c_ (0°), implies a small out‐of‐plane magnetic anisotropy energy.^[^ ^28^ ^]^ Conversely, if the magnetic anisotropy were large compared to the magnetostatic energy, e.g. in 3.5 ML Co/Au(332)^[^ ^29^ ^]^ or Iron‐9,10‐dicyanoanthracene (DCA)/Au(111),^[^ ^7^ ^]^ only the projection of the field on the magnetization direction is effective. In that case, H_c_ (60°) can be up 2 H_c_ (0°).

### 2.6 Magnetic Ordering Temperature

**Figure 3:**
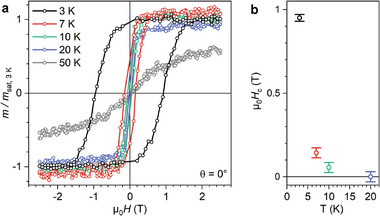
Temperature‐dependent magnetic hysteresis curves of 2D Ni‐TCNE/Au(111). a) XMCD magnetization curves recorded in normal incidence (θ = 0°) at a field sweep rate of 0.75 T min^−1^. b) Coercive fields H_c_ as a function of temperature T. At 10 K, the hysteresis loop is still open (non‐zero coercive field Hc). At 20 K there is no discernable hysteresis.

The magnetic ordering temperature is determined from temperature‐dependent magnetization curves (**Figure** **3**). At 10 K, the hysteresis curve is still slightly open (μ_0_ H_c_ = 55 ± 30 mT) but at 20 K no opening can be detected. Hence the critical temperature is in the interval 10 K < T_c_ < 20 K. At 20 K and above, single‐layer 2D Ni‐TCNE is superparamagnetic, i.e., the ferromagnetic interactions still favor parallel spin alignment, but they cannot stabilize magnetic ordering.

### 2.7 Potential Bipolar Magnetic Material

Spin polarized density functional theory (DFT) calculations with Hubbard U term have been previously performed for single‐layer 2D Ni‐TCNE in two polymorphs: i) a‐Ni‐TCNE in which all TCNE molecules exhibit alternating alignment, and ii) p‐Ni‐TCNE, where all molecules are aligned in parallel.^[^ ^30^, ^31^ ^]^ In addition to correctly predicting a 3d^9^ electronic configuration of Ni and a ferromagnetic spin alignment, it is found that 2D Ni‐TCNE is either a bipolar magnetic semiconductor (a‐Ni‐TCNE) or a bipolar magnetic metal (p‐Ni‐TCNE). In both cases, *bipolar* means that the density of states (DOS) above and below the Fermi level is fully polarized in the opposite spin‐direction. This means that the application of a gate voltage or doping affects the magnetic properties and/or spin‐transport. Interestingly, our experimentally realized 2D material consists of both a‐ and p‐polymorphs arranged in a non‐periodic configuration (Figure 1g). Therefore, its electronic properties can be expected to lie in between those predicted by theory. However, non‐periodic arrangements are also expected to show novel, exotic properties in the (spin‐resolved) density of states or (spin)‐transport.^[^ ^31^ ^]^ While an experimental validation of such predicted special electronic structure, spin‐polarization, and (spin)‐transport properties has to be yet realized, the observation that STM at a low bias voltage of 1.5 mV resolves the original lowest unoccupied molecular orbital (LUMO) of TCNE (Figure 1h) supports the theoretical prediction that said orbital is essentially singly occupied and hence found at or very close to the Fermi level.^[^ ^30^, ^32^ ^]^

### 2.8 Exchange Mechanism and Comparison with Existing Single‐Layer Low‐Dimensional Metal Organic Materials

DFT in absence of a substrate predicts significant ferromagnetic coupling through TCNE ligand orbitals.^[^ ^30^ ^]^ According to DFT, the ferromagnetic spin alignment is favored due to tunneling coupling between the doubly occupied Ni d_xz_, d_yz_ orbitals and the singly occupied former LUMO of TCNE. The observation of the LUMO at 1.5 mV in STM strongly suggests that this is also the case in the experimentally realized Ni‐TCNE layer on Au(111). Furthermore, the good agreement (structure, 3d occupation, spin in an in‐plane orbital, parallel spin alignment) between DFT^[^ ^30^ ^]^ and the present results on a gold substrate, strongly suggests that even without taken the substrate into account, the DFT results are applicable here. Nevertheless, a contribution from Ruderman–Kittel–Kasuya–Yosida (RKKY) interaction through electrons in the Au(111) substrate^[^ ^33^, ^34^ ^]^ cannot be firmly ruled out. Compared to 2D Ni‐TCNQ that is merely superparamagnetic,^[^ ^19^, ^20^, ^21^ ^]^ 2D Ni‐TCNE is a ferromagnet with a high remanent magnetization and coercive field. Its Curie temperature and coercive field are lower than the ones reported for 2D Fe‐DCA^[^ ^7^ ^]^ but higher than in case of low‐dimensional Eu‐COT.^[^ ^4^ ^]^

## 3 Conclusion

It is surprising that single‐layer transition‐metal TCNE systems have not yet been synthesized and tested for their magnetic properties. The prototypical nature of bulk TCNE organic magnets, along with the superparamagnetism observed in 2D Ni‐TCNQ^[^ ^19^, ^21^ ^]^ and Mn‐TCNB^[^ ^22^ ^]^ combined with the predicted ferromagnetic ordering in 2D Ni‐TCNE,^[^ ^30^ ^]^ strongly suggested that such investigations would be valuable. This report finally demonstrates ferromagnetism in the ordered state in a 2D TCNX (TCNQ, TCNE, TCNB) material. Specifically, 2D Ni‐TCNE exhibits at 3 K both a remarkably high magnetic remanence of 93% of the saturation magnetization and a high coercive field of ≈1 tesla. Open magnetic hysteresis is observed at least up to 10 K. XAS/XMCD/XLD, combined with multiplet calculations, reveal a 3d^9^ electronic configuration. Surprisingly, the impressive magnetic properties emerge *despite* the material having only a weak out‐of‐plane easy axis of magnetization. Scanning probe characterization reveals that Ni‐TCNE forms a square 2D lattice (a = 0.67 nm) embedding two orientations of the TCNE molecule in the lattice unit seemingly at random. This arrangement makes the single layer essentially non‐periodic.

Based on DFT, 2D Ni‐TCNE is expected to be a bipolar magnetic metal or semiconductor.^[^ ^30^ ^]^ This implies that it should be possible to electrically control the magnetic properties as well as the spin‐transport. Although the here presented characterization on a conductive Au(111) surface cannot unambiguously prove these predicted electronic properties and spin transport effects, the observation of a TCNE orbital essentially at the Fermi level is a strong indication that 2D Ni‐TCNE is indeed bipolar. Moreover, considering the Mermin‐Wagner theorem,^[^ ^35^ ^]^ that formally forbids stable magnetic order in low dimensional systems without the presence of anisotropy, the observed magnetic properties – despite the low magnetic anisotropy – provide new insights into low‐dimensional magnetism. The intriguing observation that the single‐layer 2D ferromagnet can be magnetically saturated and switched at ≈1 tesla, regardless of the field direction, is highly advantageous for spintronic applications. Finally, single‐layer metalorganic magnets have the advantages of requiring only small amounts of materials. Moreover, their properties can be tuned by choice and modification of the ligand molecule. We envision their potential as active elements in information processing and storage devices, applicable to both classical and quantum technologies.

## 4 Experimental Section

### Sample Preparation and On‐Surface Synthesis

All experiments were conducted under ultra‐high vacuum conditions (pressure p < 10^−9^ mbar). The Au(111) single crystal was cleaned by repeated cycles of Ar^+^ sputtering at 1.5 kV, followed by annealing to 500 °C. TCNE is a powder with sufficient vapor pressure to be sublimed by mild heating. Caution: TCNE forms toxic HCN with moisture from air. TCNE was dosed the vapor through a leak valve from a sealed and evacuated glass container kept at 60 °C after cleaning by pumping cycles with a turbomolecular pump.

The multilayer of TCNE on Au(111) was obtained by condensing TCNE (partial pressure 1 × 10^−7^ mbar) on the substrate kept at 110 K. Single‐layer 2D Ni‐TCNE was obtained by depositing Ni atoms from a high temperature cell (Createc, Al_2_O_3_ crucible) at an operating temperature of 1150 °C in TCNE vapor on the sample kept at 70 °C. Subsequent annealing up to 100 °C was performed to enhance the long‐range order of the MOF. Since TCNE desorbs from Au(111) at room temperature, the coverage of Ni‐TCNE is solely determined by the amount of Ni sublimed. Unreacted TCNE desorbs. The coverage of Ni‐TCNE in the XAS/XMCD experiment was determined to 0.74 monolayers (ML) by the room temperature STM of the beamline. 1 ML corresponds to a complete layer where 2nd layer growth would start. A representative image is shown in Figure S2 (Supporting Information).

### X‐ray Photoelectron Spectroscopy

XPS was measured using Al K_α_ X‐rays (1486.7 eV), in normal emission with the sample kept at room temperature. The binding energy scale was calibrated using the Au 4f_7/2_ peak (84.0 eV) and the Fermi energy (0.0 eV).

### Scanning Probe Microscopy

STM was measured using electrochemically etched and in situ sputtered tungsten tips. Low temperature STM/AFM (Createc) was performed using a QPlus sensor with a PtIr tip. Imaging parameters are reported in Table S1 (Supporting Information). The room temperature STM images were obtained using the Aarhus 150 from Specs. At the synchrotron the samples were checked using the Omicron VTSTM (Figure S2, Supporting Information).

### X‐ray Absorption Spectroscopy

XAS/XMCD/XLD experiments were conducted at the EPFL/PSI X‐Treme beamline at the Swiss Light Source^[^ ^36^ ^]^ in total electron yield mode. Circularly polarized (σ_+_, σ_−_) and linearly polarized (σ_v_, σ_h_) X‐rays were used, with the magnetic field applied co‐linear to the X‐ray beam. Background spectra recorded on the clean substrate were subtracted. The spectra were normalized to unity at the pre‐edge. The XMCD and XLD spectra represent the differences (σ_+_ – σ_−_) and (σ_v_ – σ_h_), respectively.

### Multiplet Calculations

The multiplet simulations were performed using multiX^[^ ^27^ ^]^ for 3d^9^ and 3d^8^ electronic configurations using identical crystal‐field parameters (see Table S2, Supporting Information). The simulations based on the 3d^9^ electronic configuration reproduce the experimental data well (XAS, XMCD, and XLD line shapes at L_3_ and L_2_ edges, presence of strong XMCD in both normal and grazing incidence). The simulations performed with a 3d^8^ electronic configuration fail to reproduce the experimental data.

### XMCD Sum Rule Analysis

The XMCD sum rule analysis (Figure S4, Supporting Information)^[^ ^37^, ^38^, ^39^ ^]^ was performed with a number of holes n_h_ = 1 (for the 3d^9^ electronic configuration), yielding the effective spin and orbital moment projections <S_z,eff_> and <L_z_>. It is well known that the effective values obtained from the sum rules require correction.^[^ ^37^ ^]^ Because at the Ni L_3,2_ edges there is only very low mixing of 2p_3/2_ and 2p_1/2_ components, effectively only effect of the magnetic dipole operator <T_z_> needs to be corrected.^[^ ^37^, ^40^ ^]^ Since the used multiplet code multiX^[^ ^27^ ^]^ considers both effects, the comparison of the sum‐rule results applied to the simulated spectra (<S_z,eff_>) with the calculated true values (<S_z_>), one can directly obtain the angle‐dependent correction factors c_S_ = <S_z,eff_>/<S_z_> (Table S3, Supporting Information) to determine the true spin moments from the experimental data.^[^ ^41^ ^]^

### Brief Summary of Supplementary Characterization

Figure S1 (Supporting Information) shows the Fourier transform of the Ni‐TCNE 2D crystal, confirming strong 2D crystallinity. Figure S2 (Supporting Information) provides STM images of a 0.74 ML Ni‐TCNE sample on Au(111) (presented in Figure 2), supporting single‐layer formation. Figure S3 (Supporting Information) presents multiplet calculations comparing experimental and simulated XAS/XMCD/XLD spectra for 3d⁹ and 3d⁸ configurations, validating electronic structure analysis. Figure S4 (Supporting Information) shows an example of background‐subtracted x‐ray spectra at the Ni edge, with integrals used for sum‐rule analysis. N K edge XAS that probes the unoccupied density of states of TCNE (Figure S5, Supporting Information) serves as supplementary characterization. The data shows a very strong polarization that suggests the planar orientation of the TCNE molecules on Au(111), consistent with previous reports on Ni‐TCNQ on Au(111).^[^ ^19^ ^]^ Another important finding is that Ni impurities on Au(111) (Figures S6 and S7, Supporting Information) exhibit a distinctly different XAS line shape, with the L_3_ edge appearing at a lower photon energy and demonstrating non‐magnetic behavior at the low coverage used in this study. Thus, while the SPM and XPS data provide no evidence of unreacted Ni impurities, their absence can be directly confirmed from the XAS data alone. Importantly, this ensures that the observed magnetic properties arise exclusively from the 2D Ni‐TCNE layer. Finally, Figure S8 (Supporting Information) shows magnetization curves of 2D Ni‐TCNE/Au(111) recorded at 3 K over the complete field range (±6.8 T), confirming that 2D Ni‐TCNE is completely magnetically saturated once the hysteresis opening closes at ∼ ±1 T.

### Statistical Analysis

Outliers around in magnetization curves (Figures 2d and 3a; Figure S8, Supporting Information) around zero magnetic field due to strong variation of the total electron yield are suppressed. The error bars represent 95% confidence intervals. The errors bars of the spin and orbital moments (Table 1) are determined by varying the backgrounds subtracted from the spectra, needed to determine the integrals of L_3_ and L_2_ XAS/XMCD in the sum rule analysis (cf. Figure S4, Supporting Information).

## Conflict of Interest

The authors declare no conflict of interest.

## Supporting information



Supporting Information

## Data Availability

The data that support the findings of this study are openly available in Zenodo at https://doi.org/10.5281/zenodo.10977168, reference number 10977169.
