# Supplementary material for: 2D Metalorganic Ferromagnets
Source: Adv Sci (Weinh). 2025 Mar 5;12(16):2415266. doi: 10.1002/advs.202415266 (PMC12021047; doi:10.1002/advs.202415266)
Supplement: Supplementary file 1 — Supporting Information [file ADVS-12-2415266-s001.pdf]

ADVANCED SCIENCE
Open Access

Supporting Information

for *Adv. Sci.*, DOI 10.1002/advs.202415266

2D Metalorganic Ferromagnets

Egzona Isufi Neziri, Céline Hensky, Hien Quy Le, Diego Radillo Ochoa, Aleksandra Cebrat, Manfred Parschau, Karl-Heinz Ernst and Christian Wäckerlin**

Supporting Information

Two-Dimensional Metalorganic Ferromagnets

Egzona Isufi Neziri, Céline Hensky, Hien Quy Le, Diego Radillo Ochoa, Aleksandra Cebrat, Manfred Parschau, Karl-Heinz Ernst, Christian Wäckerlin**

SPM imaging parameters

Table S1. SPM imaging parameters.

Figure	Temperature	Imaging mode and parameters
1e	4.5 K	Constant current<br>• Setpoint: 50 pA<br>• Bias voltage : 1000 mV
1f,g	300 K	Constant current<br>• Setpoint: 150 pA<br>• Bias voltage : 856 mV
1h,i,j	4.5 K	Constant height with concurrently recorded current and frequency shift<br>• Bias voltage: 1.5 mV<br>• AFM amplitude: 50 pm<br>• Current image: 4 pA (black) to 203 pA (white)<br>• Frequency shift image: −20 Hz (black) to 0 Hz (white)
S2	300 K	Constant current<br>• Setpoint: 50 pA<br>• Bias voltage : 1000 mV

2D crystallinity of NiTCNE

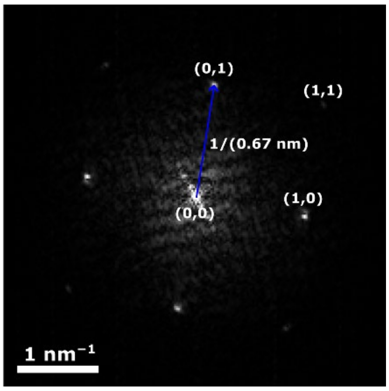

Figure S1. Fourier transform of the Ni-TCNE 2D crystal in the center of Figure 1e. The sharp peaks in reciprocal space confirm the particularly good 2D crystallinity of the lattice.

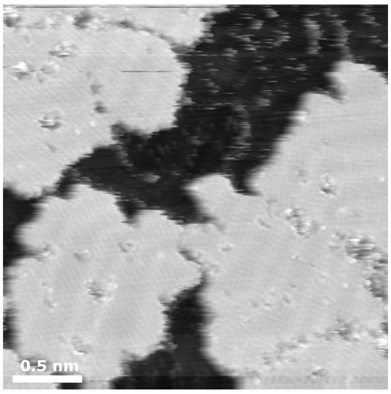

Figure S2. STM image of Ni-TCNE sample on Au(111). XAS/XMCD was measured on this sample. The coverage, based on multiple images, is 0.74 ML.

Multiplet calculations and sum-rule analysis

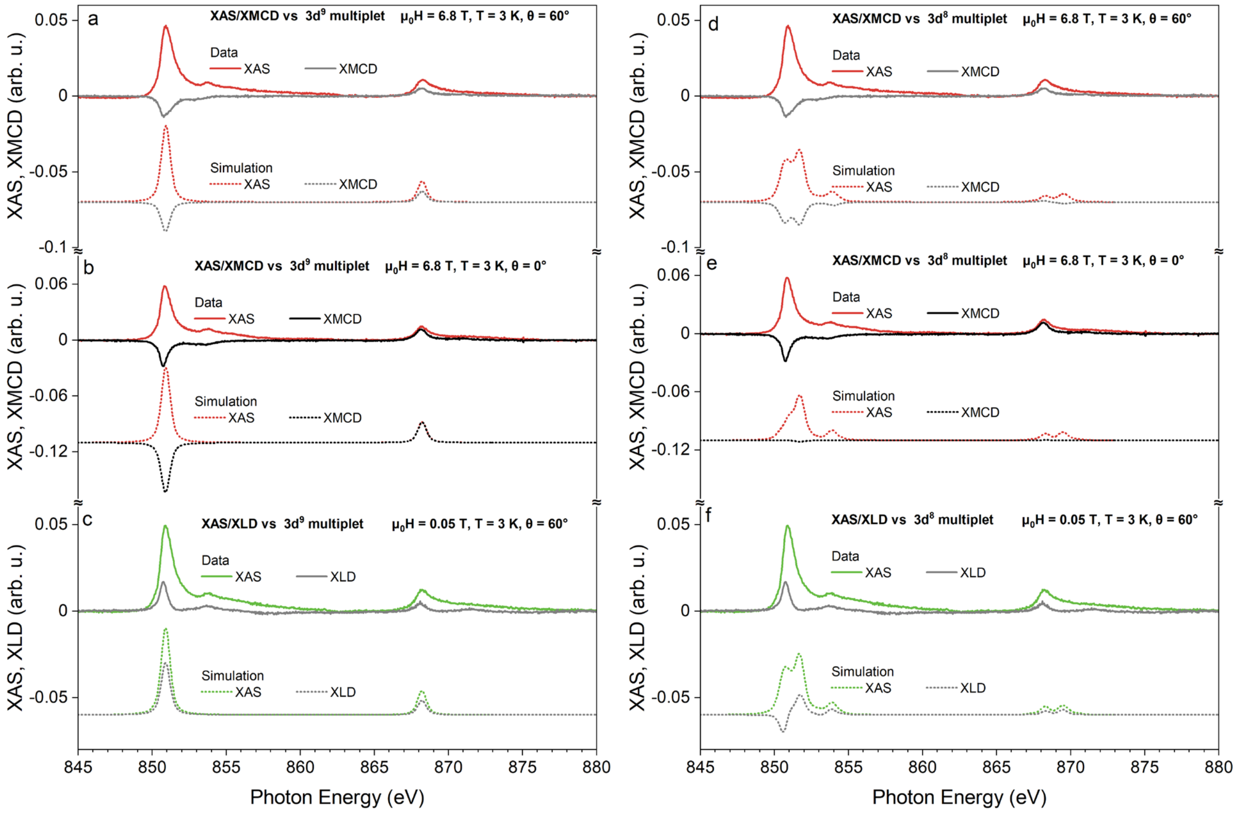

Figure S3: Multiplet calculations. Grazing incidence XAS/XMCD (a, d) normal incidence XAS/XMCD (b, e) and grazing incidence XAS/XLD data (c, f) of Ni-TCNE/Au(111) compared with simulated spectra for $3d^9$ (a-c) and $3d^8$ (d-f) electronic configurations. XAS corresponds to the sums ($\sigma_+ + \sigma_-$) and ($\sigma_v + \sigma_h$), respectively. XMCD and XLD correspond to the differences ($\sigma_+ - \sigma_-$) and ($\sigma_v - \sigma_h$), respectively.

Table S2. Real space crystal field coordinates used for the multiplet simulations. The calculated spectra were corrected by a constant energy offset, the value of the coulomb interaction was scaled to 80% of its computed value. The spin-orbit coupling was scaled to 95% ($3d^9$) and 91% ($3d^8$) of its computed value to reproduce the energy splitting between the L_3 and L_2 edges. The atomic coordinates were taken from DFT calculations.[1]

X (Å)	Y (Å)	Z (Å)	Charge (e)	Atom type
1.2717	1.5016	0	-2.0	NC
2.0257	2.3921	0	1.0	CN
2.78	3.5715	0	0.5	CC
4.227	3.5715	0	0.5	CC
4.9813	2.3921	0	1.0	CN
5.7353	1.5016	0	-2.0	NC
7.007	0	0	0.0	Ni
2.0257	4.7509	0	1.0	CN
1.2717	5.6414	0	-2.0	NC
0	7.143	0	0.0	Ni
4.9813	4.7509	0	1.0	CN
5.7353	5.6414	0	-2.0	NC
7.007	7.143	0	0.0	Ni
-5.7353	-5.6414	0	-2.0	NC
-4.9813	-4.7509	0	1.0	CN
-4.227	-3.5715	0	0.5	CC
-2.78	-3.5715	0	0.5	CC
-2.0257	-4.7509	0	1.0	CN
-1.2717	-5.6414	0	-2.0	NC
0	-7.143	0	0.0	Ni
-4.9813	-2.3921	0	1.0	CN
-5.7353	-1.5016	0	-2.0	NC
-7.007	0	0	0.0	Ni
-2.0257	-2.3921	0	1.0	CN
-1.2717	-1.5016	0	-2.0	NC
-7.007	-7.143	0	0.0	Ni
-5.7353	1.5016	0	-2.0	NC
-4.9813	2.3921	0	1.0	CN
-4.227	3.5715	0	0.5	CC
-2.78	3.5715	0	0.5	CC
-2.0257	2.3921	0	1.0	CN
-1.2717	1.5016	0	-2.0	NC
-4.9813	4.7509	0	1.0	CN
-5.7353	5.6414	0	-2.0	NC
-7.007	7.143	0	0.0	Ni
-2.0257	4.7509	0	1.0	CN
-1.2717	5.6414	0	-2.0	NC
1.2717	-5.6414	0	-2.0	NC
2.0257	-4.7509	0	1.0	CN
2.78	-3.5715	0	0.5	CC
4.227	-3.5715	0	0.5	CC
4.9813	-4.7509	0	1.0	CN
5.7353	-5.6414	0	-2.0	NC
7.007	-7.143	0	0.0	Ni
2.0257	-2.3921	0	1.0	CN
1.2717	-1.5016	0	-2.0	NC

4.9813	-2.3921	0	1.0	CN
5.7353	-1.5016	0	-2.0	NC

Table S3. Expectation values of the ground state spin and orbital moments <S_z> and <L_z> and of the effective spin and orbital moments <$S_{z,eff}$> and <$L_{z,eff}$> obtained by application of the sum-rules to the simulated spectra. The correction factor c_S relates the experimentally accessible effective spin moment <$S_{eff,z}$> obtained via sum-rule analysis to the true spin moment <S_z>.

X-ray incidence angle	Ground state of multiplet		Sum-rules applied to the calculated spectra		Correction factor
	<S_z>	<L_z>	2<$S_{z,eff}$>	<$L_{z,eff}$>	c_S = <$S_{eff,z}$> / <S_z>
**0°**	0.499	0.626	0.930	0.205	0.932
**60°**	0.470	0.250	0.516	0.125	0.549

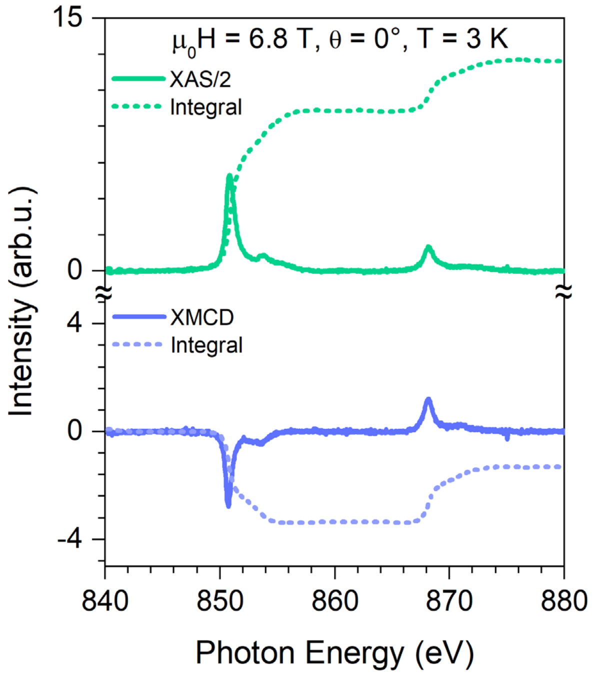

Figure S4. Example background subtracted x-ray spectra recorded at Ni $L_{2,3}$ edge along with the corresponding integrals relevant for the sum-rule analysis. For the sum rule analysis the isotropic absorption XAS/2 = ($\sigma_+ + \sigma_-$)/2 is used.

Details on the sum-rule analysis and on the strongly anisotropic spin dipole moment

Experimentally, the spin and orbital magnetic moments can be determined by application of the sum-rules[2,3] on the spectra. However, the such obtained effective spin moment <$S_{z,eff}$> can be affected by i) spectral overlap as well as by the presence of ii) a strongly anisotropic spin dipole moment <T_z>.[3] Because the used multiplet code multiX considers both effects, the most

straightforward approach is to apply the sum-rules to the simulated spectra, yielding $\langle S_{z,eff} \rangle$ and correction factor $c_S = \langle S_{eff,z} \rangle / \langle S_z \rangle$ that accounts for both effects.[4] The angle dependent corrections factors are then used to correct the experimentally determined effective spin moments.

Indeed, inspection of the sum rule-results of the simulated spectra (Table S3) yields $\mathbf{c_S(60°)} = \langle \mathbf{S_{eff,z}} \rangle / \langle \mathbf{S_z} \rangle = 0.549$, i.e. $\langle \mathbf{S_{eff,z}} \rangle$ underestimates the true spin by nearly a factor of 2. In contrast, in normal incidence this factor is close to unity. Since in case of the late transition metals (Ni, Cu) the spectra overlap is insignificant,[2] the correction factor is essentially attributed to the intra-atomic spin dipole operator $\langle T_z \rangle$. Note that these findings are in very good agreement with the previously reported multiplet analysis of Cu-Pc, a $3d^9$ case with a rather similar, square-planar crystal field[5] as well as with previously report XAS/XMCD data on Ni-TCNQ/Ag(100).[6]

Note that the fact that Ni-TCNE is fully magnetically saturated at 6.8 T in both grazing and normal incidence (Figure S8), presents the opportunity to test the validity of the above-described correction. Indeed, such obtained the experimental spin moments in normal and grazing incidence are quite similar: considering the uncertainty, the ratio $\langle S_z \rangle(0°)/\langle S_z \rangle(60°) = 0.88 \pm 0.1$ is close to unity, as expected in case of magnetic saturation.

Supplementary XAS/XMCD data

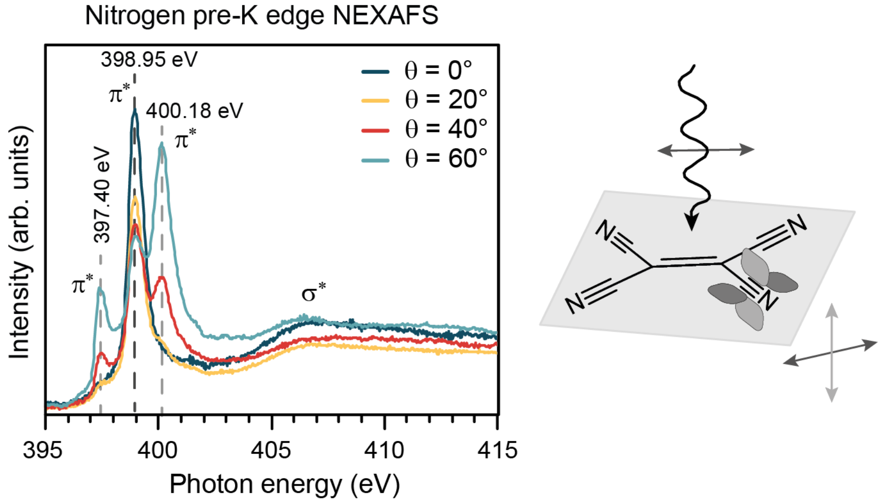

Figure S5. N K edge XAS of 2D Ni-TCNE on Au(111) taken at incidence angles θ ranging from 0° to 60° ($\mu_0 H$ = 50 mT; T = 300 K, polarization σ_h, *i.e.* nearly out-of-plane at θ = 60°). Very similar N K edge spectra were reported for Ni-TCNQ.[6] In principle, the split of the π*

resonance can be understood by hybridization the of one of the C≡N π-bonds with the central C=C π-bond.[7,8]

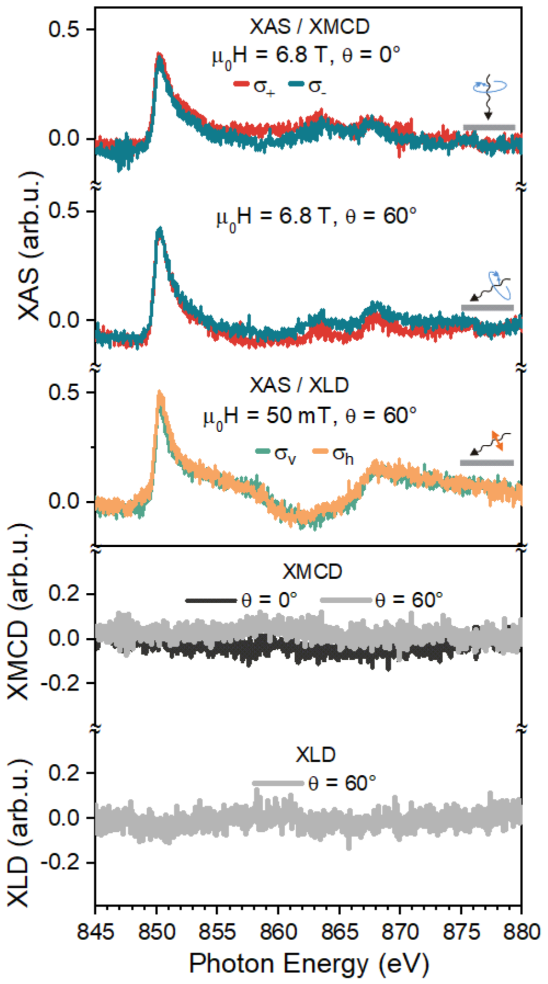

Figure S6. XAS/XMCD/XLD characterization of metallic, uncoordinated nickel on/in Au(111). The sample was made by deposition of nickel without TCNE molecules. The spectra do not exhibit any XMCD signal even at high applied magnetic field (6.8 T) and low temperature (3 K), i.e. the nickel is non-magnetic. Most importantly, the XAS line-shapes are very different than the ones of Ni-TCNE and there is no XLD signal.

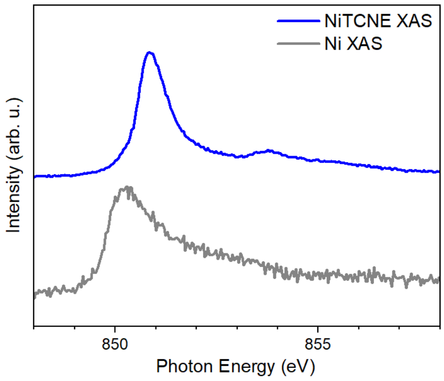

Figure S7. Ni L_3 edge XAS of Ni-TCNE compared with deliberately made metallic nickel on Au(111). As expected, it presents a broader, edge-like line shape like metallic nickel, while Ni in Ni-TCNE exhibits a much narrower XAS peak at an increased photon energy, reminiscent transition-metal elements in oxides or molecules.

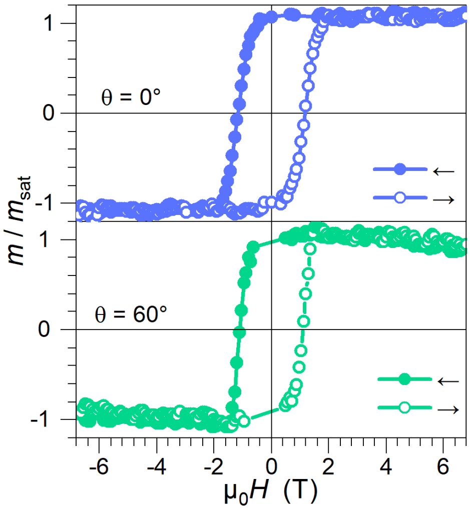

Figure S8: Magnetization curves of 2D Ni-TCNE/Au(111) recorded at 3 K over the complete field range (±6.8 T). The field sweep rate is 2.0 T/min. The data confirms that Ni-TCNE is completely magnetically saturated once the hysteresis opening closes at ~ ±1 T.

References

[1] Y. Chen, J. Liu, Q. Sun, Y. Kawazoe, P. Jena, *Adv. Electron. Mater.* **2018**, *4*, 1700323.
[2] P. Carra, B. Thole, M. Altarelli, X. Wang, *Phys. Rev. Lett.* **1993**, *70*, 694.
[3] C. Piamonteze, P. Miedema, F. M. F. de Groot, *Phys. Rev. B* **2009**, *80*, 184410.
[4] C. Wäckerlin, F. Donati, A. Singha, R. Baltic, A.-C. Uldry, B. Delley, S. Rusponi, J. Dreiser, *Chem. Commun.* **2015**, *51*, 12958.
[5] S. Stepanow, A. Mugarza, G. Ceballos, P. Moras, J. C. Cezar, C. Carbone, P. Gambardella, *Phys. Rev. B* **2010**, *82*, 014405.
[6] N. Abdurakhmanova, T.-C. Tseng, A. Langner, C. S. Kley, V. Sessi, S. Stepanow, K. Kern, *Phys. Rev. Lett.* **2013**, *110*, 027202.
[7] R. Sumii, K. Amemiya, *Journal of Electron Spectroscopy and Related Phenomena* **2010**, *182*, 51.
[8] J. Stöhr, *NEXAFS Spectroscopy*, Springer, Berlin, **1996**.
